# Anatomical location-based nodal staging system is superior to the 7^th^ edition of the American Joint Committee on Cancer staging system among patients with surgically resected, histologically low-grade gastric cancer: A single institutional experience

**DOI:** 10.1371/journal.pone.0211836

**Published:** 2019-02-05

**Authors:** Mei-Wen Chen, Chien-Pin Chan, Yih-Jeng Lin, Hsu-Heng Yen

**Affiliations:** 1 Department of Tumor Center, ChangHua Christian Hospital, ChangHua, Taiwan; 2 Department of Information Management,Chien-Kuo Technology University, ChungHua, Taiwan; 3 Department of General Surgery, ChangHua Christian Hospital, ChangHua, Taiwan; 4 Department of Gastroenterology, ChangHua Christian Hospital, ChangHua, Taiwan; 5 General Education Center, Chien-Kuo Technology University, ChungHua, Taiwan; Bilkent University, TURKEY

## Abstract

**Background:**

A hybrid topographic and numeric lymph node (LN) staging system for gastric cancer, which was recently proposed by Japanese experts as a simple method with a prognostic predictive power comparable to the N staging of the American Joint Committee on Cancer (AJCC) Tumor-node-metastasis classification, has not yet been validated in other Asian countries. This study aimed to examine the prognostic predictability of the hybrid staging system with the current AJCC staging system in gastric cancer.

**Methods:**

Overall, 400 patients with gastric cancer who underwent surgery at Changhua Christian Hospital from January 2007 to December 2017 were included in the study. Univariate and multivariate analyses were performed to identify prognostic factors for gastric cancer-related death. Homogeneity and discrimination abilities of the two staging systems were compared using likelihood ratio chi-square test, linear trend chi-square test, Harrell’s c-index, and bootstrap analysis.

**Results:**

One-third of the LN-positive patients were reclassified into the new N and Stage system. The concordance rates of the two staging systems and the N staging between the two staging systems were 0.810 and 0.729, respectively. Harrell’s c-indices for the stage and N staging were higher in the 7^th^ AJCC staging system than the hybrid staging system (c-index for stage, 0.771 vs 0.764; c-index for nodal stage, 0.713 vs 0.705). Stratification of the patients according to the histological grade revealed that Harrell’s c-indices for the stage and N stage of the hybrid staging system were comparable with those of the 7^th^ AJCC staging system (c-index for AJCC stage vs hybrid stage, 0.800 vs 0.791; c-index for AJCC N stage vs hybrid N stage, 0.746 vs 0.734) among patients with histologically lower grade gastric cancer. The performance of the new nodal staging system was better than that of the 7^th^ AJCC staging system by likelihood ratio and linear trend tests and bootstrap analysis in the low-grade group.

**Conclusions:**

The hybrid anatomical location-based classification may have better prognostic predictive ability than the 7^th^ AJCC staging system for LN metastasis of low-grade gastric cancer. Further studies involving different ethnic populations are necessary for the validation of the new staging system.

## Introduction

Gastric cancer incidence rates vary wildly between males and females and across different countries.[1, 2] The lifetime risk of gastric cancer is higher in Japan, Korea, and India; intermediate at approximately 3% in Eastern Europe, South America, and certain regions in Asia including parts of China and the Golestan Province in Iran; and low in other countries.[1, 2] The tumor-node-metastasis (TNM) classification and staging system of the American Joint Committee on Cancer (AJCC) is the most important reference for accurate and reproducible staging of gastric cancer in daily practice, and periodic and reasonable revisions of the TNM staging system are made by the Union for International Cancer Control and the AJCC.[3, 4] More than half of patients with gastric cancer have lymph node (LN) metastasis at diagnosis or after surgery, with poor prognosis.[5] Before its 5^th^ edition, nodal (N) staging within the TNM classification was based on the anatomical extent of LN metastasis.[6] Starting with the 5^th^ AJCC edition, N staging has been based on the number of involved LNs[7, 8] and the anatomic extent of LN metastasis is no longer included. Unlike tumor (T) staging within the TNM classification, which relies on pathological assessment, harvesting LNs for N staging relies on surgical techniques.[9] The numeric N staging does not offer information on the anatomical extent of disease and does not represent the quality of LN dissection.[9, 10] For instance, D2 lymphadenectomy with dissection of perigastric and extraperigastric LNs, which is usually performed in the East and has been recently recommended in the West, provides more information on metastatic LNs compared with D1 resection.[11] Using data from Japan and Korea, Choi *et al*.[10] proposed a hybrid LN staging system, which demonstrated a prognostic performance equal to that of the 7^th^ edition of the TNM system. This new and simple staging system was proposed to be a reliable alternative to the current numeric-based system in an Italian study.[12]

In this study, we compared the performance of the new N staging proposed by Choi *et al*.[10] and the 7^th^ edition of the AJCC staging system based on data from 400 Taiwanese patients with surgically treated gastric cancer at a single institution. We reclassified the cohort participants based on the new N staging to reach a new staging system we termed as the hybrid anatomy-based staging system and compared the predictive ability and prognostic performance of the two staging systems.

## Methods

### Patients

Patient data between January 2007 and December 2017 were obtained from the cancer registry database of Changhua Christian Hospital located in central Taiwan and reviewed and approved by a committee of oncologists, radiotherapists, nurse specialists, surgeons, and pathologists. This retrospective study was approved by the Institutional Review Board of Changhua Christian Hospital (approval number: 170907). Among a total of 1325 patients with gastric cancer who underwent surgical intervention in the Department of Surgery at Changhua Christian Hospital (the 3^rd^ edition of the International Classification of Diseases code C161–C169), 925 (69.81%) patients were excluded because of (1) primary tumor location in cardia (n = 80), (2) surgery not performed (n = 443), (3) pathology other than adenocarcinoma (n = 191 cases), (4) presence of preoperative chemotherapy (n = 12), (5) surgery performed outside of a hospital (n = 94), (6) missing or incomplete data for T or N staging (n = 28), and (7) diagnosis of multiple cancers (n = 77). Finally, 400 patients were included in the study. Baseline data included demographic characteristics, including sex and age, and tumor characteristics including grade, tumor depth of invasion, regional LNs, pathologic staging per the 7^th^ AJCC edition, and the new N staging. All patients were followed until March 15, 2018. Postoperative follow-up was for a minimum of 3–6 months for physical and laboratory evaluations and annually thereafter for up to 10 years after surgery or until death.

### Reclassification based on the new N system

The patients were categorized based on the anatomical location of LNs into the perigastric and extraperigastric LN groups.^(13)^ The perigastric LN group was divided further into lesser curvature (LN groups 1, 3, and 5) and greater curvature (LN groups 2, 4sa, 4sb, 4d, 6, and greater omentum) groups. The patients with LNs in other anatomical locations were classified as the extraperigastric group. In the new N staging, Choi *et al*.(10) classified the LN status into four categories: 1) new N0, indicated by no metastatic LNs in any group; 2) new N1, indicated by one positive LN among the three groups (positive LC alone, positive GC alone, or positive EP alone), regardless of the number; 3) new N2, indicated by two positive LNs out of the three groups (positive LC + GC, positive LC + EP, or positive GC + EP), regardless of the number; and 4) new N3, defined as positive LNs in all three groups (LC + GC + EP) (Table 1).

**Table 1:** Comparison of lymph classification between AJCC 7th system and Anatomical location-based nodal staging system.

N Stage	AJCC 7^th^	Anatomical location-based nodal staging system
N0	0	0
N1	1–2 lymph nodes	Involvement one of LC/GC/EP Groups
N2	3–6 lymph nodes	Involvement two of LC/GC/EP Groups
N3	≥7 lymph nodes	Involvement all of LC/GC/EP Groups

Abbrevations for Table 1. LC Group: lesser curvature lymph node group. GC Group: greater curvature lymph node group. EP: Group: extra-perigastric lymph node group

### Statistical analysis

Majority of the statistical analyses were performed using SPSS statistical software (version 22.0; SPSS, Chicago, IL, USA). *P* values less than.05 were considered statistically significant. The Kaplan-Meier method was used to analyze time-dependent survival probabilities, and the log-rank test was used for statistical comparisons of survival curves. Overall survival was calculated from the day of diagnosis to the date of death or last follow-up. Date of death due to other causes was obtained from the databases of the tumor cancer registry as censored.

The Cox proportional hazards model was used to evaluate the risk of mortality associated with the prognostic factors selected in the multivariate analysis of clinical parameters. Prognostic homogeneity was tested by the likelihood ratio chi-square test, and the linear trend chi-square analysis was used to assess the discrimination ability of the different editions of the staging system in patients with gastric cancer. A higher likelihood ratio chi-square value indicated better homogeneity of the staging scheme, and a higher linear trend chi-square likelihood ratio indicated a better discriminatory ability. Kappa values were determined to evaluate the degree of conformity between the two staging systems. To compare the prognostic performance of the staging systems, Harrell’s c-index was used to measure their predictive accuracy in survival outcomes.[13,14] The SAS statistical software (SAS Institute, Cary, NC, USA) was used to compare the prognostic performance of the hybrid anatomy-based staging and the 7^th^ edition of the AJCC staging for low- and high-grade cancers by bootstrap analysis, and mean values of differences with 95% confidence intervals were determined.

## Results

### Demographic data and survival of the patients

**Fig 1:**
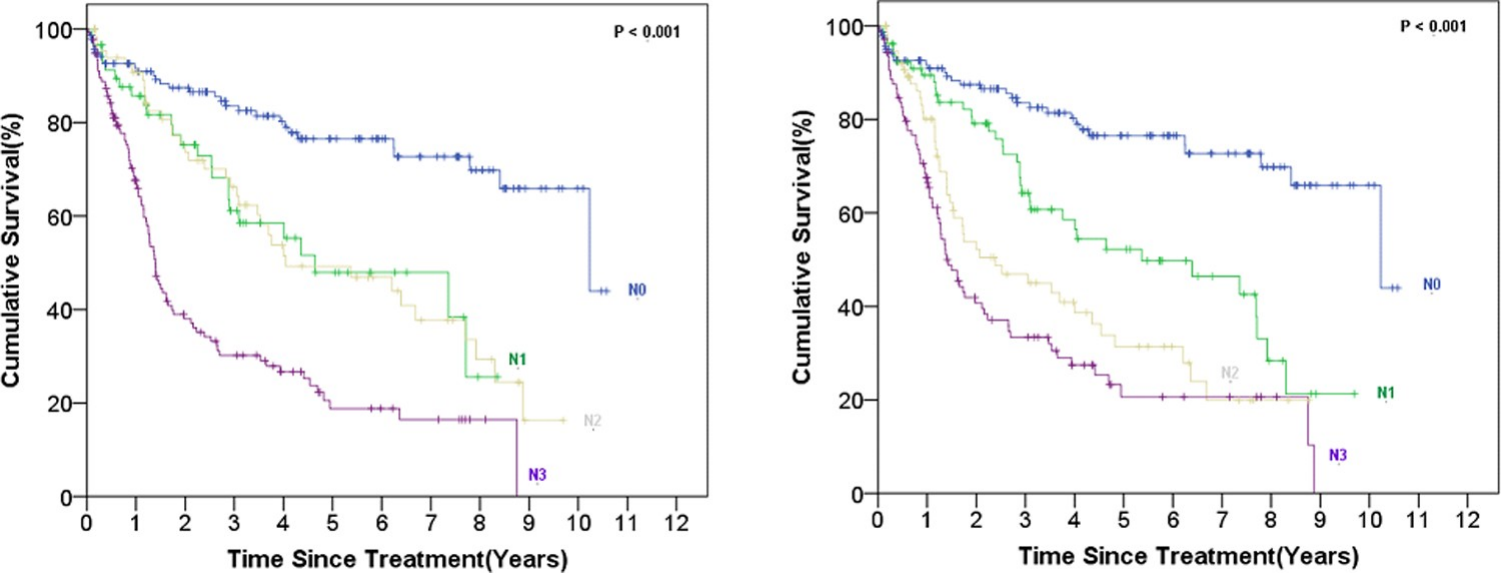
The survival curves according to the N status based on the 7^th^ AJCC staging system (left) and the new hybrid anatomy-based system (right).

**Fig 2:**
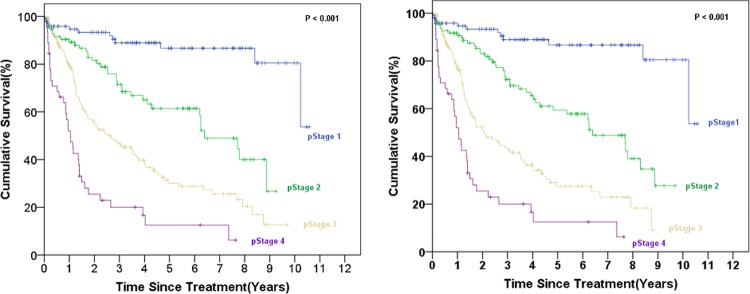
The survival curves according to the stage based on the 7^th^ AJCC staging system (left) and the new hybrid anatomy-based system (right).

The demographic and clinical characteristics of 400 patients who underwent surgical resection for gastric cancer and met the inclusion criteria of the study are provided in Table 2. The study included 248 (62%) males and 152 (38%) females. The mean age at diagnosis was 66.24 ± 12.29 (range, 24–96) years. The tumor depth of invasion was T1A, T1B, T2, T3, T4A, and T4B in 30 (7.5%), 50 (12.5%), 57 (14.25%), 137 (34.25%), 105 (26.25%), and 21 (5.25%) patients, respectively. The tumor histological grade was low (G1/G2) and high (G3) in 112 (28%) and 288 (72%) patients, respectively. The number of retrieved LNs was more than 27 in 256 (64%) of the patients. The univariate analysis revealed that patient age, tumor primary site, tumor depth of invasion, N status based on the 7^th^ AJCC staging system, stage according to the 7^th^ AJCC staging system, new LN position, and new stage system were significantly associated with 5-year-survival. The 5-year-survival curves using the 7^th^ AJCC staging system and the new system (N and Stage status) are illustrated in Figs 1 and 2.

**Table 2:** Characteristics and overall survival rate of patients with gastric cancer.

Variables	Patients (%)	5 y OS (%)	mean OS (95% CI)	*P*
**Age (years), mean** ± **SD, range**	66.24 ± 12.29 (24–96)			.001
**Sex**				.588
Man	248 (62.00)	47.576	5.801 (4.998–6.604)	
Female	152 (38.00)	50.102	5.228 (4.674–5.782)	
**Primary Site**				.001
C161, fundus of stomach	7 (1.75)	0.000	2.404 (1.121–3.686)	
C162, body of stomach	93 (23.25)	51.745	6.073 (5.046–7.100)	
C163, gastric antrum	221 (55.25)	52.191	5.759 (5.127–6.391)	
C164, pylorus	32 (8.00)	46.276	4.172 (3.165–5.179)	
C165, lesser curvature of stomach, NOS	17 (4.25)	71.500	6.535 (4.744–8.326)	
C166, greater curvature of stomach, NOS	2 (0.50)	0.000	0.697 (0.000–1.715)	
C168, overlapping lesion of stomach	28 (7.00)	14.286	5.510 (5.028–5.992)	
**Grade**				.616
G1, well; G2, moderately differentiated	112 (28.00)	46.829	5.110 (4.293–5.927)	
G3, poorly differentiated/undifferentiated	288 (72.00)	49.029	5.573 (5.006–6.139)	
**T (Tumor depth of invasion)**				.001
1A, lamina propria or muscularis mucosae	30 (7.50)	95.000	9.718 (8.705–10.731)	
1B, submucosa	50 (12.50)	80.862	8.563 (7.438–9.689)	
2, muscularis propria	57 (14.25)	64.798	6.729 (5.587–7.871)	
3, subserosa	137 (34.25)	47.064	5.060 (4.364–5.756)	
4A, serosa (visceral peritoneum)	105 (26.25)	20.287	3.070 (2.398–3.742)	
4B, adjacent structures	21 (5.25)	14.435	2.261 (0.998–3.523)	
**AJCC 7**^**th**^ **edition (regional lymph nodes)**				.001
0	139 (34.75)	76.514	8.136 (7.411–8.861)	
1, 1–2	58 (14.50)	47.923	4.969 (4.048–5.890)	
2, 3–6	68 (17.00)	49.265	5.178 (4.273–6.083)	
3, ≥7	135 (33.75)	18.827	2.847 (2.270–3.425)	
**AJCC 7**^**th**^ edition, TNM stage				.001
1A	68 (17.00)	90.368	9.452 (8.680–10.224)	
1B	30 (7.50)	78.923	8.405 (7.059–9.750)	
2A	52 (13.00)	57.992	5.651 (4.592–6.710)	
2B	43 (10.75)	65.514	6.118 (5.028–7.208)	
3A	55 (13.75)	41.586	4.906 (3.922–5.889)	
3B	50 (12.50)	33.726	4.170 (3.196–5.145)	
3C	57 (14.25)	12.558	2.349 (1.471–3.223)	
4	45 (11.25)	12.529	1.935 (1.202–2.669)	
**New** system, TNM stage				.001
1A	68 (17.00)	90.368	9.452 (8.680–10.224)	
1B	30 (7.50)	78.923	8.405 (7.059–9.750)	
2A	56 (14.00)	56.103	5.478 (4.463–6.494)	
2B	53 (13.25)	62.225	6.387 (5.363–7.412)	
3A	51 (12.75)	39.563	4.334 (3.383–5.284)	
3B	48 (12.00)	26.861	3.719 (2.685–4.753)	
3C	49 (12.25)	13.138	2.494 (1.569–3.419)	
4	45 (11.25)	12.529	1.935 (1.202–2.669)	
No. of resected lymph nodes				.834
≤27	144 (36.00)	50.443	5.667 (4.840–6.495)	
>27	256 (64.00)	47.500	5.408 (4.822–5.995)	
Lymph node position (New N staging)				.001
**new N0**	**139 (34.75)**	**76.514**	**8.136 (7.411–8.861)**	
**new N1**	**78 (19.50)**	**52.236**	**5.435 (4.565–6.036)**	
LC alone	35	57.345	6.038 (4.843–7.234)	
GC alone	31	47.285	4.671 (3.408–5.934)	
EP alone	12	53.571	4.746 (2.861–6.632)	
**new N2**	**77 (19.25)**	**31.393**	**3.747 (2.937–4.557)**	
LC + GC	31	46.316	4.289 (2.914–5.665)	
LC + EP	20	23.325	3.213 (1.855–4.572)	
GC + EP	26	20.380	3.348 (2.228–4.469)	
**new N3**	**106 (26.50)**	**20.647**	**3.056 (2.364–3.749)**	
LC + GC + EP	106	20.647	3.056 (2.364–3.749)	
**Perigastric LN**	97	50.250	5.187 (4.373–6.000)	
**Extra-perigastric LN**	163	23.622	3.316 (2.762–3.870)	

### The relationship between the new staging system and the AJCC 7th system

Table 3 illustrates the distribution of patients based on N staging using the number of regional metastatic LNs according to the 7^th^ AJCC and the hybrid staging systems. There were 139 (34.75%), 58 (14.5%), 68 (17%), and 135 (33.75%) patients in stages N0, N1, N2, and N3, respectively, according to the 7^th^ AJCC staging system. Conversely, there were 139 (34.75%), 78 (19.5%), 77 (19.25%), and 106 (26.5%) patients in stages N0, N1, N2, and N3, respectively, according to the new hybrid N staging system. Accordingly, 10 of the 58 patients (17.24%) in stage N1 of the 7^th^ AJCC staging system were classified as new stage N2 of the hybrid system. Among the 68 patients in stage N2 according to the 7^th^ AJCC staging system, 26 (38.24%) and 7 (10.29%) patients were reclassified as new stages N1 and N2, respectively. Furthermore, among the 135 patients in stage N3 according to the 7^th^ AJCC staging system, 4 (2.96%) and 32 (23.70%) patients were reclassified as new stages N1 and N2, respectively. The kappa values for determining the degree of conformity between the 7^th^ AJCC staging system and the new hybrid system for the stage and the N stage were 0.810 and 0.729, respectively.

**Table 3:** The distribution of patients according to the 7^th^ AJCC staging system and the new hybrid staging system.

		**TNM 7**^**th**^ **edition, pStage**								
		**1A**	**1B**	**2A**	**2B**	**3A**	**3B**	**3C**	**4**	**Total**
**New pStage**	**1A**	68	0	0	0	0	0	0	0	**68**
	**1B**	0	30	0	0	0	0	0	0	**30**
	**2A**	0	0	49	7	0	0	0	0	**56**
	**2B**	0	0	3	32	18	0	0	0	**53**
	**3A**	0	0	0	4	29	16	2	0	**51**
	**3B**	0	0	0	0	8	33	7	0	**48**
	**3C**	0	0	0	0	0	1	48	0	**49**
	**4**	0	0	0	0	0	0	0	45	**45**
	**Total**	**68**	**30**	**52**	**43**	**55**	**50**	**57**	**45**	**400**
Kappa = 0.810										
	**TNM 7**^**th**^ **edition, pN**									
		**0**		**1**		**2**		**3**		**Total**
**New pN**	**0**	139		0		0		0		**139**
	**1**	0		48		26		4		**78**
	**2**	0		10		35		32		**77**
	**3**	0		0		7		99		**106**
	**Total**	**139**		**58**		**68**		**135**		**400**
Kappa = 0.729										

Computed by inter-rater agreement analysis and kappa value, where a kappa value of 0.20 indicates poor agreement and a kappa value of 0.80 indicates very good agreement. Concordance between the 7^th^ AJCC stage and the new hybrid stage, kappa = 0.810. Concordance between the current N stage and new N stage, kappa value = 0.729.

### Comparison of the prognostic performance between the new hybrid staging system and the 7^th^ AJCC staging system

The prognostic performance of the 7^th^ AJCC and the hybrid staging systems were compared using Harrell’s c-index (Table 4), linear likelihood ratio, and linear trend tests. Harrell’s c-index, indicating the prognostic performance, was comparable for the N and Stage between the 7^th^ AJCC and the hybrid staging systems (pN, 0.713 and 0.705; pStage, 0.771 and 0.764, according to the 7^th^ AJCC and hybrid staging systems, respectively). The overall performance was comparable for both the N stage and the stage between the two staging systems. The likelihood ratio and the linear trend test showed similar results (Table 5). However, regarding tumor grade (low [G1/G2] vs high [G3]), we found that the hybrid staging system performed better than the 7^th^ AJCC staging system in the low-grade group (G1/ G 2) with a higher Harrell’s c-index, a high likelihood ratio, and a higher linear trend for both the N and the stage.

**Table 4:** The prognostic performance of each staging system by Harrell’s C-index.

**All Patients**	**Harrell’s C-index**	**95%CI**	**P value**
**7**^**th**^ **pN**	0.713	0.662–0.764	.001
**New pN**	0.705	0.654–0.757	.001
**7**^**th**^ **pStage**	0.771	0.725–0.816	.001
**New pStage**	0.764	0.718–0.810	.001
**G1/G2**	**Harrell’s C–index**	**95%CI**	**P value**
**7**^**th**^ **pN**	0.626	0.522–0.730	.022
**New pN**	0.639	0.535–0.742	.011
**7**^**th**^ **pStage**	0.699	0.602–0.796	.001
**New pStage**	0.716	0.621–0.812	.001
**G3**	**Harrell’s C–index**	**95%CI**	**P value**
**7**^**th**^ **pN**	0.746	0.688–0.803	.001
**New pN**	0.734	0.676–0.791	.001
**7**^**th**^ **pStage**	0.800	0.750–0.850	.001
**New pStage**	0.791	0.740–0.842	.001

CI, confidence interval

**Table 5:** Comparison of the prognostic performance between the 7^th^ AJCC staging system and the new hybrid staging system for nodal and TNM stages.

**All Patients**	**7**^**th**^ **pN**	**New pN**	**7**^**th**^ **pStage**	**New pStage**
Likelihood Ratio*	62.136	58.309	99.432	95.846
Linear Trend**	58.863	53.272	83.977	81.588
**G1/G2**	**7th pN**	**New pN**	**7th pStage**	**New pStage**
Likelihood Ratio*	6.030	9.904	15.836	18.031
Linear Trend**	5.491	6.435	9.790	10.779
**G3**	**7th pN**	**New pN**	**7th pStage**	**New pStage**
Likelihood Ratio*	61.555	56.909	91.467	88.445
Linear Trend**	57.743	49.664	77.985	73.676

*Likelihood Ratio chi-square test: higher values indicate better homogeneity (a small difference in overall survival among patients classified into the same group by the new system)

** Linear Trend chi-square test: higher values indicate better discriminatory power (patients classified into different groups have greater differences in overall survival) and monotonicity.

Bootstrap analysis revealed that the performance of the new hybrid staging system was inferior to that of the 7^th^ AJCC staging system (*p* < .001) (Table 6). However, further analysis with stratification of the cohort to the low-grade and high-grade groups revealed that the new hybrid staging system had a significantly improved performance in the low-grade group.

**Table 6:** Prognostic performance between the current and new nodal and TNM stages by bootstrap analysis.

	Bootstrap analysis for N parameter*							
	**7**^**th**^ **N**		**New pN**		**Difference**			
	**Mean**	**SD**	**Mean**	**SD**	**Mean**	**SD**	**95%CI**	**P value**
**Likelihood Ratio**	106.5	18.432	102.9	18.199	−3.541	18.316	−5.148 to −1.935	<0.001
**Linear Trend**	84.322	15.023	81.981	14.915	−2.340	14.969	−3.654 to −1.028	<0.001
	Bootstrap analysis for N parameter* (G1/G2)							
	**7**^**th**^ **pN**		**New pN**		**Difference**			
	**Mean**	**SD**	**Mean**	**SD**	**Mean**	**SD**	**95%CI**	**P value**
**Likelihood Ratio**	9.087	5.452	12.790	6.241	3.703	5.860	3.189 to 4.217	0.001
**Linear Trend**	6.297	4.568	7.206	4.848	0.903	4.710	0.496 to 1.322	0.001
	Bootstrap analysis for N parameter* (G3)							
	**7**^**th**^ **pN**		**New pN**		**Difference**			
	**Mean**	**SD**	**Mean**	**SD**	**Mean**	**SD**	**95%CI**	**P value**
**Likelihood Ratio**	64.484	14.657	59.866	14.263	−4.618	14.461	−5.886 to −3.450	0.001
**Linear Trend**	58.424	12.950	50.378	12.310	−8.046	12.634	−9.154 to −6.938	0.001
	Bootstrap analysis for TNM stage							
	**7**^**th**^ **TNM stage**		**New TNM stage**		**Difference**			
	**Mean**	**SD**	**Mean**	**SD**	**Mean**	**SD**	**95%CI**	**P value**
**Likelihood Ratio**	64.922	15.105	61.204	14.723	−3.718	14.915	−5.026 to −2.410	<0.001
**Linear Trend**	59.451	13.942	53.981	13.504	−5.470	13.725	−6.673 to −4.266	<0.001
	Bootstrap analysis for TNM stage (G1/G2)							
	**7**^**th**^ **TNM stage**		**New TNM stage**		**Difference**			
	**Mean**	**SD**	**Mean**	**SD**	**Mean**	**SD**	**95%CI**	**P value**
**Likelihood Ratio**	23.505	8.831	25.590	9.092	2.086	8.962	1.299 to 2.872	0.001
**Linear Trend**	10.4739	5.785	11.400	5.882	0.924	5.834	0.4121 to 1.435	0.001
	Bootstrap analysis for TNM stage (G3)							
	**7**^**th**^ **TNM stage**		**New TNM stage**		**Difference**			
	**Mean**	**SD**	**Mean**	**SD**	**Mean**	**SD**	**95%CI**	**P value**
**Likelihood Ratio**	58.424	12.950	50.378	12.310	−8.046	12.634	−9.154~−6.938	0.001
**Linear Trend**	98.174	16.674	95.242	16.502	−2.932	16.588	−4.386~−1.477	0.001

*1000 samples

## Discussion

In this retrospective study, we found that the hybrid anatomy-based staging system provided a better overall prognostic stratification than the 7^th^ AJCC staging system in patients with lower histological grade gastric cancer. In contrast with the previous studies by Choi *et al*.[10] and Gennaro *et al*.,[12] both of which suggested that the hybrid anatomy-based staging system was better than the currently utilized 7^th^ AJCC staging system, our findings suggest that the tumor histological grade might play an important role and should be considered in this hybrid anatomy-based staging system to improve the homogeneity and the discriminatory ability of the current AJCC staging system.

The AJCC TNM staging system is the global standard that guides clinical decision-making and prognostic prediction. Because of the geographic differences in incidence and mortality gastric cancer[13,14] and variations in surgical techniques, the 7^th^ AJCC staging system based on data mainly from the US may be valid for a particular population[15,16] but not equally applicable to other ethnic groups.[7, 17] The extent of LN metastasis was proven to be an important independent prognostic factor in gastric cancer[18–20] with the evolution of the N subset of the AJCC staging system. Compared with the previous editions, the 7^th^ edition strengthened the role of the number of positive LNs by subdividing the LN classes into 0, 2, 6 or more rather than the anatomical LN distribution. Several studies[8, 21] suggested that the N classification of the 7^th^ edition was not superior to the previous editions in evaluating the prognostic relevance of LN status, which may be related to the surgical techniques using limited lymphadenectomy (D1 lymphadenectomy) and the fewer number of LNs retrieved[8] in Western countries. With the trend to perform extended lymphadenectomy with more LNs harvested, patient outcomes have improved,[11, 19, 22] and more information regarding the LN status can be obtained for prognostic stratification of patients.[19]

The anatomical information on LN metastasis in gastric cancer is important but not convenient for clinical use.[23] The hybrid approach utilized for LN staging by Choi *et al*^.^[10] using data from Japan and Korea was equivalent to the 7^th^ AJCC staging system in prognostic performance, suggesting the importance of the anatomical distribution of metastatic LNs.[6] The new N system utilized the anatomical involvement of LNs to categorize into the LC, GC, and EP groups, which is more straightforward than counting the number of retrieved LNs and the number of metastatic LNs utilized by the AJCC staging system (Table 1). Considering the anatomical distribution of LN involvement is more straightforward than counting the number of retrieved LNs in the daily practice (Table 1). For example, if the patient had three regional lymph node involvement would be classified as N2 in the AJCC 7^th^ system. The patient would be classified as N1 if all the nodes were belonged to same anatomical groups or N3 if they belonged to three different anatomical groups in the new anatomical location-based nodal staging. The different distribution of lymph node involvement may reflect the different behavior of the tumor and influence the performance of staging system. The current study comparing the performance of the proposed hybrid staging system and the 7^th^ AJCC staging system in patients with gastric cancer revealed that the hybrid staging system performed better only in patients with histologically low-grade cancer, suggesting that tumor histology may be important for prognostic stratification. The proportion of patients with high-grade cancer in the current cohort (72%) was different than those in studies from China (71.2%),[23] Korea (61%),[6] and Italy (40%).[12] A difference in the proportion of cancers based on tumor histology was reported to be associated with a difference in the pattern of LN metastasis,[24, 25] which may explain the inconsistent prognostic performance of different staging systems for gastric cancer.

The AJCC staging system is based on only clinical parameters, i.e., tumor size, LN status, and metastasis; thus, it may not perfectly reflect complex real-world patients with gastric cancer. Approaches for LN retrieval and evaluation are still evolving for the treatment of gastric cancer.[19] The number of retrieved LNs is associated with survival for LN-negative gastric cancer.[26, 27] Several modifications to the LN staging system, such as log odds of positive LNs,[28, 29] LN ratio,[30] and anatomical LN distribution,[23] were proposed to improve the AJCC N system. A modified T classification of gastric cancer into proximal non-diffuse, diffuse, and distal non-diffuse types based on Lauren's classification of gastric cancer was reported.[31, 32] The recent hybrid LN staging system[33] involves the least change to the AJCC system and is simple and attractive for clinical use. A Western study from Italy[12] including 284 gastric cancer patients suggested that the new hybrid classification was significantly correlated with tumor recurrence rate and displayed improved indices of prognostic performance. The current study results suggest that the new hybrid staging system might be applicable only for patients with histologically low-grade cancer, suggesting the need for consideration of the tumor biology[34] for further development of prognostic systems for specific patient populations.

The current study has several limitations. This was an observational study, and selection bias could not be denied. Additionally, not all patients with gastric cancer treated during the study period were included, mainly because of incomplete pathology records or limited follow-up information. The sample size was relatively small, and the study included one institution. Major strengths of the current study were that most of the surgeries were performed by two expert surgeons and the patients were managed by a multidisciplinary team at a hospital with uniform surgical techniques and postoperative care; however, additional larger-scale studies are still necessary before reaching stronger conclusions.

## Conclusion

Despite its simplicity, the current study results suggest that the hybrid anatomy-based staging system developed by Choi *et al*.^(10)^ exhibited improved performance only for patients with histologically low-grade gastric cancer. Additional studies with larger sample sizes and different ethnic populations should compare the performance of the new anatomy-based LN stating system with the existing AJCC staging system.

## Supporting information

S1 File The original dataset for analysis. (PDF)

## Abbreviations

AJCC: American Joint Committee on Cancer
LN: Lymph node
SAS: Statistical software
TNM: Tumor-node-metastasis
LC: lesser curvature
GC: greater curvature
EP: extra-perigastric
