# Supplementary material for: Anatomical location-based nodal staging system is superior to the 7th edition of the American Joint Committee on Cancer staging system among patients with surgically resected, histologically low-grade gastric cancer: A single institutional experience
Source: PLoS One. 2019 Feb 5;14(2):e0211836. doi: 10.1371/journal.pone.0211836 (PMC6363228; doi:10.1371/journal.pone.0211836)
Supplement: S1 File — (PDF) [file pone.0211836.s001.pdf]

id	age	primary si	OS	ydtime	SEX	sexcode	grade	group7nan	group7nn	group4nn
1	58	C163	1	1.73	M	1	2	LC	1	1
2	85	C168	1	1.04	F	0	4	LCGCEP	7	3
3	82	C163	0	1.08	M	1	4	LCEP	5	2
4	78	C163	0	2.18	M	1	2	GC	2	1
5	68	C162	0	2.82	M	1	4		0	0
6	62	C163	0	4.38	M	1	4	LCGCEP	7	3
7	60	C163	0	1.53	F	0	4	LCGC	4	2
8	52	C162	0	10.58	F	0	4		0	0
9	81	C162	1	8.30	M	1	4	LC	1	1
10	57	C163	0	4.45	M	1	4		0	0
11	65	C163	0	0.98	F	0	4	LCGCEP	7	3
12	85	C164	1	0.05	F	0	4		0	0
13	79	C163	0	4.37	M	1	4		0	0
14	62	C163	1	1.43	M	1	4	LCGCEP	7	3
15	65	C164	0	4.47	F	0	4		0	0
16	64	C162	1	1.40	M	1	4	LCGCEP	7	3
17	66	C163	0	0.53	M	1	2		0	0
18	80	C162	1	3.10	F	0	2	LC	1	1
19	60	C165	0	8.04	F	0	4		0	0
20	75	C164	1	1.21	M	1	2	LCGCEP	7	3
21	67	C163	1	3.70	F	0	4	GCEP	6	2
22	70	C162	1	0.33	M	1	2		0	0
23	79	C163	1	0.50	M	1	4	LCGCEP	7	3
24	62	C162	0	1.06	M	1	4		0	0
25	79	C163	1	0.02	M	1	2	LCGCEP	7	3
26	85	C162	1	0.09	M	1	2		0	0
27	67	C165	1	3.09	M	1	2	LC	1	1
28	61	C162	0	0.98	F	0	4	LCEP	5	2
29	83	C163	1	0.54	M	1	4	LCGCEP	7	3
30	77	C162	0	5.48	M	1	4	LC	1	1
31	82	C163	0	7.13	M	1	4		0	0
32	76	C162	1	1.61	M	1	2	LCGCEP	7	3
33	84	C163	1	0.22	F	0	4	LCGCEP	7	3
34	81	C165	0	1.75	F	0	4		0	0
35	83	C163	1	0.08	M	1	4	LCGCEP	7	3
36	71	C163	1	1.29	M	1	4	LCGCEP	7	3
37	85	C162	0	0.86	F	0	4		0	0
38	63	C162	0	6.33	F	0	4		0	0
39	82	C162	1	1.17	M	1	2	EP	3	1
40	79	C164	0	3.10	F	0	4		0	0
41	76	C163	0	6.32	M	1	4		0	0
42	69	C163	1	4.42	M	1	4	LCGCEP	7	3
43	71	C163	1	1.90	F	0	4	LC	1	1
44	73	C163	0	0.39	F	0	2		0	0
45	70	C163	1	4.69	F	0	4	LCGCEP	7	3
46	62	C163	0	8.47	M	1	4		0	0
47	81	C163	1	0.79	F	0	4	LCGC	4	2
48	75	C163	1	1.75	M	1	4	LCEP	5	2
49	79	C163	0	2.73	F	0	4		0	0
50	70	C163	1	1.39	F	0	4	LCGC	4	2

51	47	C163	0	0.16	F	0	4		0	0
52	70	C163	0	2.80	F	0	4		0	0
53	55	C163	0	5.95	F	0	4		0	0
54	76	C163	0	8.27	F	0	2		0	0
55	69	C162	0	0.11	F	0	4		0	0
56	79	C163	1	2.88	M	1	4	EP	3	1
57	72	C163	0	3.22	M	1	2	GCEP	6	2
58	86	C163	1	0.13	M	1	4	GC	2	1
59	77	C164	1	6.39	F	0	4	GC	2	1
60	58	C163	0	2.83	M	1	4		0	0
61	80	C163	1	1.65	F	0	2	LCGCEP	7	3
62	67	C163	0	4.71	M	1	4	LCGCEP	7	3
63	75	C162	1	0.05	M	1	4		0	0
64	55	C163	1	0.41	F	0	4	GCEP	6	2
65	36	C163	0	9.25	M	1	4		0	0
66	60	C163	0	7.52	F	0	4		0	0
67	78	C162	0	4.18	F	0	4		0	0
68	71	C163	0	10.10	F	0	4		0	0
69	68	C163	1	7.92	M	1	4	GC	2	1
70	68	C163	1	0.48	F	0	4	LCGCEP	7	3
71	63	C163	1	1.50	M	1	2	LCGCEP	7	3
72	76	C163	1	2.88	M	1	2	GC	2	1
73	54	C168	1	0.06	F	0	4	LCGCEP	7	3
74	53	C162	0	8.81	M	1	2	LC	1	1
75	66	C161	1	1.37	F	0	4	LCGCEP	7	3
76	53	C163	1	1.26	F	0	4	LCEP	5	2
77	58	C163	0	1.87	M	1	2		0	0
78	61	C163	0	7.58	F	0	2		0	0
79	67	C163	0	2.25	M	1	2	LC	1	1
80	80	C162	0	6.77	M	1	4		0	0
81	84	C163	1	10.23	F	0	4		0	0
82	76	C163	0	1.22	F	0	2		0	0
83	67	C162	0	10.47	F	0	4		0	0
84	79	C163	0	8.24	M	1	4	GC	2	1
85	74	C168	1	2.09	M	1	2		0	0
86	69	C162	1	1.73	M	1	4	LCEP	5	2
87	73	C163	1	1.91	M	1	4	GC	2	1
88	69	C163	0	7.66	M	1	4	GC	2	1
89	55	C163	0	5.92	M	1	2		0	0
90	58	C163	0	5.48	M	1	4		0	0
91	60	C163	0	2.44	M	1	4		0	0
92	81	C164	1	0.20	M	1	2	GCEP	6	2
93	60	C163	1	0.93	M	1	2	LCGC	4	2
94	59	C162	1	1.16	F	0	4	GCEP	6	2
95	75	C163	1	2.90	M	1	2	LC	1	1
96	96	C162	1	0.17	M	1	2	LCGC	4	2
97	49	C162	0	7.83	F	0	4		0	0
98	61	C162	0	2.62	M	1	4	LCEP	5	2
99	75	C162	0	0.23	F	0	4		0	0
100	76	C163	0	2.17	F	0	2		0	0
101	45	C163	0	1.96	F	0	4	EP	3	1

102	66	C163	1	1.25	F	0	4	LCGCEP	7	3
103	66	C168	1	0.23	M	1	4	LCGCEP	7	3
104	46	C163	0	9.68	F	0	4		0	0
105	74	C163	0	4.07	M	1	4	LC	1	1
106	53	C163	1	1.14	F	0	4	GC	2	1
107	67	C162	1	1.72	M	1	2	LCGC	4	2
108	67	C168	1	2.25	M	1	4	GC	2	1
109	78	C164	1	3.53	M	1	2	GCEP	6	2
110	85	C163	0	0.16	M	1	4	LCEP	5	2
111	73	C168	1	1.61	F	0	4	LCGCEP	7	3
112	52	C162	1	2.53	F	0	4	LCEP	5	2
113	63	C162	1	3.45	M	1	4		0	0
114	64	C162	0	3.45	M	1	4		0	0
115	71	C163	0	0.16	M	1	4	GCEP	6	2
116	83	C163	1	2.61	M	1	4		0	0
117	76	C164	0	4.93	M	1	2		0	0
118	44	C168	0	8.79	F	0	2		0	0
119	87	C163	1	4.29	M	1	4		0	0
120	68	C164	1	6.36	M	1	2	LCGC	4	2
121	71	C162	0	5.88	M	1	4		0	0
122	57	C162	1	1.20	M	1	4	GCEP	6	2
123	55	C163	0	5.98	M	1	4	LCGC	4	2
124	74	C163	1	0.15	M	1	2		0	0
125	75	C163	0	3.18	M	1	4	LCGCEP	7	3
126	75	C162	1	1.22	M	1	4	LCGCEP	7	3
127	49	C163	0	0.15	F	0	4		0	0
128	56	C163	0	6.80	F	0	4	LC	1	1
129	67	C162	0	1.48	M	1	4	LC	1	1
130	78	C163	0	2.93	M	1	2	GC	2	1
131	75	C163	1	0.18	F	0	4	GC	2	1
132	66	C162	0	0.28	M	1	4		0	0
133	63	C163	0	2.11	F	0	4		0	0
134	43	C163	0	1.10	F	0	4	GCEP	6	2
135	43	C163	0	0.87	F	0	4		0	0
136	79	C162	0	2.42	M	1	4		0	0
137	89	C163	1	0.65	M	1	2	GCEP	6	2
138	81	C162	1	2.39	M	1	4	LC	1	1
139	81	C163	1	2.39	F	0	4	LCEP	5	2
140	73	C168	1	0.59	F	0	4	LCGCEP	7	3
141	60	C162	0	8.11	M	1	2	LCGCEP	7	3
142	71	C163	0	8.51	F	0	2		0	0
143	49	C163	0	7.15	F	0	4	LCGCEP	7	3
144	59	C162	0	9.70	M	1	2	LC	1	1
145	73	C168	1	1.10	F	0	4	LCGCEP	7	3
146	80	C163	0	4.36	M	1	4	LCGCEP	7	3
147	70	C163	0	3.25	M	1	4	LCGCEP	7	3
148	79	C165	0	8.91	M	1	2	LC	1	1
149	63	C162	1	4.54	F	0	4	GCEP	6	2
150	52	C163	0	6.27	F	0	4	EP	3	1
151	73	C163	0	2.41	M	1	4		0	0
152	71	C161	1	1.46	M	1	4	GCEP	6	2

153	77	C163	0	5.50	M	1	4	LCGC	4	2
154	68	C162	0	0.39	M	1	2		0	0
155	54	C163	0	5.07	M	1	2		0	0
156	57	C163	0	8.05	M	1	4		0	0
157	57	C164	1	0.91	M	1	4	LCEP	5	2
158	64	C163	0	0.09	M	1	4		0	0
159	54	C162	0	8.77	F	0	4	LCGC	4	2
160	71	C168	1	0.95	M	1	4	LCGCEP	7	3
161	75	C164	0	0.29	M	1	2	LC	1	1
162	69	C162	1	1.40	M	1	2		0	0
163	71	C163	0	9.34	M	1	2		0	0
164	37	C163	0	7.54	F	0	4		0	0
165	83	C163	1	4.36	M	1	4	GCEP	6	2
166	68	C163	0	8.35	F	0	4	LCEP	5	2
167	55	C163	0	3.52	M	1	2	LC	1	1
168	55	C168	1	1.28	M	1	4	LCGCEP	7	3
169	69	C163	0	5.78	M	1	4	LC	1	1
170	77	C163	1	4.82	F	0	4	LCEP	5	2
171	79	C162	1	0.37	M	1	2	GC	2	1
172	88	C168	1	0.13	F	0	2		0	0
173	53	C163	0	4.32	M	1	2		0	0
174	67	C163	0	3.12	M	1	2	LC	1	1
175	59	C165	1	0.42	F	0	4	LCEP	5	2
176	72	C162	1	0.59	M	1	4	LCGCEP	7	3
177	72	C163	1	0.68	F	0	4	LCGCEP	7	3
178	31	C163	1	3.76	M	1	4	LC	1	1
179	73	C163	1	0.32	M	1	4	GC	2	1
180	85	C163	1	2.54	M	1	2	GC	2	1
181	69	C163	0	3.53	M	1	4	EP	3	1
182	73	C168	1	2.16	M	1	4	LCGCEP	7	3
183	56	C164	1	1.17	M	1	2	GCEP	6	2
184	65	C163	0	9.60	F	0	4		0	0
185	55	C163	1	2.65	M	1	4	LCGCEP	7	3
186	79	C168	1	2.70	M	1	4	LCGCEP	7	3
187	57	C162	0	3.80	M	1	4		0	0
188	60	C162	0	3.05	F	0	4	GC	2	1
189	69	C163	0	5.72	F	0	4	GC	2	1
190	53	C164	1	3.04	F	0	2	GCEP	6	2
191	79	C163	1	1.36	M	1	2	LCGCEP	7	3
192	67	C162	1	4.03	M	1	4		0	0
193	75	C163	1	0.11	M	1	4	GC	2	1
194	75	C162	0	0.54	F	0	4	LCGC	4	2
195	79	C168	1	0.30	F	0	4	LCGCEP	7	3
196	81	C163	0	2.35	M	1	2		0	0
197	46	C168	0	8.69	F	0	4		0	0
198	57	C168	1	0.45	F	0	4	LCGCEP	7	3
199	81	C165	0	0.72	M	1	2	LC	1	1
200	71	C166	1	1.22	M	1	4	GC	2	1
201	45	C165	0	7.45	M	1	4	LC	1	1
202	79	C163	1	6.24	M	1	4		0	0
203	72	C162	0	4.38	F	0	4		0	0

204	50 C162	1	1.40 F	0	4 LCEP	5	2
205	56 C163	1	5.37 F	0	4 LC	1	1
206	72 C163	1	0.77 M	1	4 LCGCEP	7	3
207	72 C163	1	1.28 M	1	4 LCGCEP	7	3
208	72 C163	1	0.11 M	1	2	0	0
209	33 C163	0	0.62 M	1	2 LCGC	4	2
210	56 C163	0	4.17 M	1	4	0	0
211	48 C163	0	3.79 M	1	4 LCGC	4	2
212	56 C163	0	3.63 M	1	4 LCGCEP	7	3
213	63 C163	1	0.98 M	1	4	0	0
214	82 C163	1	0.77 F	0	4 LCGCEP	7	3
215	40 C164	0	0.82 M	1	4	0	0
216	76 C162	0	0.53 M	1	4 GCEP	6	2
217	34 C163	0	0.37 F	0	4	0	0
218	54 C163	0	0.09 M	1	4	0	0
219	64 C163	1	7.72 M	1	4 LC	1	1
220	81 C163	1	3.94 F	0	4 LCGCEP	7	3
221	57 C163	0	3.45 M	1	4 LCGCEP	7	3
222	62 C163	0	3.18 F	0	4 GC	2	1
223	82 C163	1	0.81 F	0	4 LCGCEP	7	3
224	52 C162	0	5.05 F	0	4 EP	3	1
225	62 C162	0	7.64 M	1	4 GCEP	6	2
226	80 C164	0	7.55 F	0	2	0	0
227	72 C163	1	2.00 F	0	4 LCGC	4	2
228	66 C168	1	0.95 F	0	4 LCGCEP	7	3
229	63 C163	1	4.95 M	1	2 LCGCEP	7	3
230	64 C168	0	3.94 M	1	4 LCGCEP	7	3
231	82 C163	0	0.92 F	0	4 LCGCEP	7	3
232	56 C163	0	0.16 F	0	4 LCGC	4	2
233	82 C163	0	0.13 M	1	4 LCGCEP	7	3
234	54 C161	1	4.01 M	1	2 LC	1	1
235	71 C163	1	0.58 M	1	2 GCEP	6	2
236	68 C163	0	2.32 F	0	4 LCGCEP	7	3
237	58 C163	1	0.66 F	0	2 LC	1	1
238	60 C164	0	0.94 M	1	4 GC	2	1
239	56 C163	0	7.58 M	1	4 LCGC	4	2
240	77 C163	1	2.24 F	0	4 LCGCEP	7	3
241	59 C163	1	0.37 F	0	4 LCGCEP	7	3
242	61 C162	0	0.48 M	1	4 LCEP	5	2
243	71 C163	1	2.92 M	1	4 LC	1	1
244	45 C162	0	3.97 F	0	4	0	0
245	76 C162	1	4.12 M	1	4	0	0
246	58 C165	0	7.80 M	1	2 LCGCEP	7	3
247	83 C163	1	2.07 F	0	2 LCGC	4	2
248	43 C162	0	0.58 F	0	4 LCGCEP	7	3
249	68 C163	0	7.27 F	0	2	0	0
250	58 C164	0	5.31 M	1	2 GCEP	6	2
251	73 C164	1	6.25 F	0	2	0	0
252	56 C163	0	4.75 M	1	4 LCGCEP	7	3
253	48 C163	0	1.97 M	1	4 LCGCEP	7	3
254	74 C163	0	2.28 M	1	4	0	0

255	71 C163	0	3.34 M	1	4	0	0
256	79 C163	0	2.36 F	0	2 LCGC	4	2
257	59 C162	1	1.01 F	0	4 LCGCEP	7	3
258	77 C164	1	0.31 M	1	2 EP	3	1
259	70 C163	0	0.18 F	0	2 LCGCEP	7	3
260	59 C165	0	0.17 F	0	4	0	0
261	55 C161	1	1.18 M	1	4 EP	3	1
262	71 C163	0	2.84 F	0	4	0	0
263	58 C162	0	5.57 F	0	4	0	0
264	56 C162	0	0.57 F	0	4	0	0
265	73 C165	1	0.23 M	1	4 LCGCEP	7	3
266	69 C163	1	3.48 F	0	4 LCGCEP	7	3
267	66 C163	1	6.69 M	1	2 LCGC	4	2
268	81 C164	1	2.55 M	1	4 EP	3	1
269	71 C163	0	7.34 M	1	4 LCGC	4	2
270	60 C163	1	1.16 M	1	4 LCGC	4	2
271	65 C163	0	5.58 M	1	2	0	0
272	63 C164	0	1.01 M	1	2 LCGCEP	7	3
273	73 C164	1	3.65 F	0	4 LCGCEP	7	3
274	58 C163	0	4.06 M	1	4	0	0
275	75 C162	1	0.51 F	0	4 LCGCEP	7	3
276	64 C163	1	0.86 F	0	4 LCGCEP	7	3
277	65 C166	1	0.18 M	1	4 LCGCEP	7	3
278	59 C161	0	1.05 M	1	2	0	0
279	63 C163	0	2.45 M	1	4	0	0
280	44 C163	0	7.91 M	1	4	0	0
281	73 C163	1	8.87 M	1	4 LCGCEP	7	3
282	86 C168	1	1.54 M	1	2 LCGC	4	2
283	42 C168	0	9.98 M	1	4	0	0
284	73 C163	0	3.24 F	0	4	0	0
285	58 C163	1	3.93 M	1	4	0	0
286	35 C163	0	6.33 F	0	4 GCEP	6	2
287	58 C163	0	8.92 M	1	4	0	0
288	57 C165	0	8.19 M	1	2	0	0
289	71 C162	0	7.78 F	0	4	0	0
290	80 C162	0	6.08 F	0	4	0	0
291	65 C162	0	7.58 M	1	4	0	0
292	50 C162	0	7.71 F	0	4 LCGCEP	7	3
293	62 C164	0	5.53 M	1	2	0	0
294	54 C163	1	0.85 F	0	4 LCGCEP	7	3
295	66 C163	1	0.88 M	1	4 LCEP	5	2
296	73 C163	0	3.05 M	1	4 LCGCEP	7	3
297	82 C168	1	0.84 F	0	4 LCGCEP	7	3
298	86 C162	0	0.65 M	1	4 LCEP	5	2
299	68 C164	0	0.87 M	1	4 LC	1	1
300	61 C165	0	0.19 M	1	2 LCGCEP	7	3
301	72 C163	0	4.61 M	1	2	0	0
302	83 C163	1	1.35 M	1	2	0	0
303	74 C163	0	3.98 F	0	4 LCEP	5	2
304	86 C162	1	1.49 M	1	2	0	0
305	51 C162	0	5.79 M	1	4 LCGCEP	7	3

306	67	C163	0	1.40	M	1	2		0	0
307	76	C165	1	7.70	M	1	2	LC	1	1
308	72	C163	1	8.76	M	1	4	LCGCEP	7	3
309	53	C163	0	7.96	M	1	2	GC	2	1
310	75	C163	1	1.01	M	1	2	LCGCEP	7	3
311	44	C168	1	0.26	M	1	4	LCGCEP	7	3
312	52	C162	1	2.65	M	1	4	LCGCEP	7	3
313	74	C163	1	1.98	M	1	4	LCGCEP	7	3
314	51	C162	0	4.27	F	0	4		0	0
315	70	C163	0	4.36	M	1	4		0	0
316	73	C163	1	1.73	M	1	4	LCGCEP	7	3
317	56	C162	0	3.95	F	0	4	LCGCEP	7	3
318	80	C162	1	0.32	M	1	4		0	0
319	84	C163	1	2.83	M	1	2		0	0
320	50	C163	0	3.10	F	0	2	LCGC	4	2
321	55	C164	0	2.06	M	1	4		0	0
322	71	C163	0	0.43	M	1	4	LCGCEP	7	3
323	64	C163	0	1.00	M	1	4	LCGCEP	7	3
324	68	C163	0	1.07	F	0	4	GC	2	1
325	36	C163	0	0.10	M	1	2		0	0
326	65	C162	1	0.14	F	0	4	LCGCEP	7	3
327	57	C163	0	0.62	M	1	2	GCEP	6	2
328	76	C163	0	5.76	M	1	2	GC	2	1
329	52	C163	0	2.21	M	1	4	GC	2	1
330	53	C165	0	8.51	M	1	4		0	0
331	69	C165	1	0.86	F	0	2	LCEP	5	2
332	76	C165	1	8.41	M	1	4		0	0
333	24	C164	1	1.67	M	1	4		0	0
334	78	C163	1	0.15	M	1	2		0	0
335	50	C163	1	2.83	M	1	4	GC	2	1
336	63	C163	0	3.27	M	1	2		0	0
337	49	C162	1	2.12	M	1	4	LCGCEP	7	3
338	77	C162	1	0.50	M	1	4	GCEP	6	2
339	78	C164	0	6.22	F	0	2	LCGCEP	7	3
340	69	C168	1	1.76	M	1	4	LCGCEP	7	3
341	74	C162	0	5.81	M	1	2		0	0
342	72	C163	1	2.73	M	1	4		0	0
343	49	C163	0	4.96	F	0	4		0	0
344	81	C164	1	1.25	F	0	4	LCGC	4	2
345	75	C164	0	2.97	F	0	2	GC	2	1
346	42	C163	1	1.41	M	1	4	GCEP	6	2
347	58	C168	1	1.15	F	0	4	LCGC	4	2
348	61	C163	1	0.14	F	0	4	LCGCEP	7	3
349	56	C163	0	2.30	M	1	4	GCEP	6	2
350	57	C168	1	0.39	M	1	4	LCGCEP	7	3
351	47	C164	0	1.64	M	1	4	LCGCEP	7	3
352	58	C162	1	0.39	M	1	4	LCGCEP	7	3
353	53	C163	0	1.20	M	1	4	LCGCEP	7	3
354	69	C163	0	0.61	F	0	4	LCGC	4	2
355	64	C162	1	4.64	F	0	2	GC	2	1
356	71	C163	1	1.11	F	0	4	LCGCEP	7	3

357	78	C163	0	4.65 M	1	2 LC	1	1
358	27	C163	0	1.30 F	0	4	0	0
359	66	C162	0	8.55 M	1	4	0	0
360	44	C163	0	6.35 F	0	4	0	0
361	77	C162	0	3.23 F	0	4 LC	1	1
362	82	C162	1	0.26 M	1	4	0	0
363	63	C163	0	1.20 F	0	4 LCGC	4	2
364	60	C168	1	0.19 M	1	4 LCGC	4	2
365	71	C163	1	6.21 M	1	4 LCEP	5	2
366	78	C163	0	5.13 M	1	4 EP	3	1
367	73	C163	1	7.79 F	0	4	0	0
368	42	C162	0	3.69 M	1	4	0	0
369	85	C162	0	6.79 F	0	4	0	0
370	77	C163	1	3.53 M	1	4 LCGCEP	7	3
371	79	C168	1	0.25 M	1	4 LCGCEP	7	3
372	71	C162	1	3.10 M	1	2	0	0
373	37	C162	0	6.02 M	1	4	0	0
374	56	C163	0	6.50 M	1	2 LC	1	1
375	45	C162	0	5.83 M	1	4 LCGC	4	2
376	51	C163	0	1.25 M	1	4 EP	3	1
377	70	C162	0	3.67 F	0	4	0	0
378	61	C163	1	1.38 F	0	4 LCGCEP	7	3
379	59	C163	0	1.04 M	1	4 LCGCEP	7	3
380	79	C162	0	0.84 F	0	2	0	0
381	78	C163	0	4.19 M	1	4 LCGCEP	7	3
382	74	C162	0	0.57 M	1	2 LCGCEP	7	3
383	53	C162	0	0.31 M	1	2 LC	1	1
384	79	C163	1	1.52 M	1	2 LCGC	4	2
385	54	C165	0	8.81 M	1	4	0	0
386	66	C163	1	0.89 F	0	4 LC	1	1
387	68	C162	1	7.36 M	1	2 EP	3	1
388	70	C163	1	1.01 M	1	2	0	0
389	45	C163	1	0.22 M	1	4 LCGCEP	7	3
390	62	C163	1	0.97 F	0	4 LCGCEP	7	3
391	50	C163	1	1.05 F	0	2 LCGCEP	7	3
392	61	C161	1	4.00 M	1	4 GCEP	6	2
393	81	C163	0	4.25 F	0	4 GCEP	6	2
394	73	C162	1	0.22 F	0	2 LCGC	4	2
395	70	C164	1	4.05 M	1	4 LC	1	1
396	66	C163	0	3.61 M	1	4	0	0
397	59	C163	0	1.41 F	0	4 LCGCEP	7	3
398	91	C163	0	1.21 F	0	4 GC	2	1
399	73	C164	0	0.61 M	1	2 GC	2	1
400	61	C161	0	0.39 M	1	2 GC	2	1

nnstage7	T7ajcc	N7ajcc	stage7ajcc	N7ajcc4	nngroup2	resectionln2
2A	2	2	2B	2	1	1
4	3	3B	4	3	2	2
3A	3	2	3A	2	2	2
2B	3	1	2B	1	1	2
1A	1B	0	1A	0	0	2
3B	3	2	3A	2	2	2
3B	4A	2	3B	2	1	2
1A	1B	0	1A	0	0	2
2B	3	2	3A	2	1	2
1A	1B	0	1A	0	0	1
3B	3	3A	3B	3	2	1
1B	2	0	1B	0	0	2
1A	1A	0	1A	0	0	1
3C	4A	3A	3C	3	2	2
1B	2	0	1B	0	0	1
4	3	3A	4	3	0	2
1A	1A	0	1A	0	0	1
2A	2	1	2A	1	1	2
2B	4A	0	2B	0	0	2
3C	4A	3B	3C	3	2	2
3B	4A	2	3B	2	2	2
2A	3	0	2A	0	0	1
3C	4A	3B	3C	3	2	2
2B	4A	0	2B	0	0	1
4	4B	3A	4	3	2	2
1B	2	0	1B	0	0	2
3A	4A	2	3B	2	1	2
2A	1A	3A	2B	3	2	1
3C	4A	3A	3C	3	2	2
2B	3	2	3A	2	1	1
1A	1B	0	1A	0	0	1
4	4A	3A	4	3	2	2
4	4A	3B	4	3	2	2
2A	3	0	2A	0	0	2
4	4A	3B	4	3	2	2
3B	3	3B	3B	3	2	2
2B	4A	0	2B	0	0	1
1A	1B	0	1A	0	0	2
3B	4B	1	3B	1	2	1
1B	2	0	1B	0	0	2
1A	1B	0	1A	0	0	1
3B	3	3B	3B	3	2	2
2B	3	2	3A	2	1	2
1A	1A	0	1A	0	0	1
3B	3	3A	3B	3	2	1
1A	1A	0	1A	0	0	1
3A	3	2	3A	2	1	2
3A	3	1	2B	1	2	2
1A	1A	0	1A	0	0	2
3B	4A	3B	3C	3	1	2

1A	1B	0	1A	0	0	2
1A	1A	0	1A	0	0	1
2A	3	0	2A	0	0	1
1A	1B	0	1A	0	0	2
1A	1A	0	1A	0	0	1
2B	3	1	2B	1	2	1
3A	3	2	3A	2	2	1
4	3	1	4	1	1	1
2A	2	2	2B	2	1	2
1A	1B	0	1A	0	0	1
3A	2	3B	3A	3	2	2
3C	4A	3A	3C	3	2	2
2A	3	0	2A	0	0	1
3C	4B	3A	3C	3	2	1
2A	3	0	2A	0	0	1
1B	2	0	1B	0	0	2
1A	1A	0	1A	0	0	2
1B	2	0	1B	0	0	2
3A	4A	2	3B	2	1	2
3B	3	3A	3B	3	2	2
3B	3	3A	3B	3	2	2
2A	2	1	2A	1	1	1
3C	4A	3B	3C	3	2	1
3A	4A	2	3B	2	1	1
3B	3	3B	3B	3	2	2
3A	3	3A	3B	3	2	2
1A	1B	0	1A	0	0	1
1B	2	0	1B	0	0	1
2B	3	2	3A	2	1	1
1B	2	0	1B	0	0	1
1A	1B	0	1A	0	0	1
2A	3	0	2A	0	0	2
1A	1A	0	1A	0	0	2
2A	2	2	2B	2	1	2
2A	3	0	2A	0	0	1
3A	3	2	3A	2	2	1
2A	2	1	2A	1	1	1
2B	3	1	2B	1	1	2
1A	1B	0	1A	0	0	1
1B	2	0	1B	0	0	2
2A	3	0	2A	0	0	1
3A	3	2	3A	2	2	1
3B	4A	2	3B	2	1	2
3C	4B	3A	3C	3	2	2
3A	4A	1	3A	1	1	1
3A	3	2	3A	2	1	1
1A	1B	0	1A	0	0	2
3A	3	3A	3B	3	2	2
1A	1A	0	1A	0	0	2
1A	1B	0	1A	0	0	1
1B	1A	1	1B	1	2	1

3B	3	3B	3B	3	2	2
4	4A	3B	4	3	2	2
1A	1A	0	1A	0	0	1
2A	2	1	2A	1	1	1
4	4A	2	4	2	1	1
3B	4A	1	3A	1	1	1
2B	3	1	2B	1	1	2
3A	3	2	3A	2	2	1
3B	4A	2	3B	2	2	1
3C	4A	3A	3C	3	2	2
2A	1B	3B	2B	3	2	2
2A	3	0	2A	0	0	2
1A	1B	0	1A	0	0	2
2B	2	2	2B	2	2	1
1B	2	0	1B	0	0	1
3B	4B	0	3B	0	0	2
2B	4A	0	2B	0	0	2
2A	3	0	2A	0	0	2
3A	3	3A	3B	3	1	1
1B	2	0	1B	0	0	1
3C	4B	2	3C	2	2	1
3A	3	3A	3B	3	1	2
1A	1B	0	1A	0	0	1
3B	3	3B	3B	3	2	2
3C	4A	3B	3C	3	2	2
1A	1A	0	1A	0	0	1
2B	3	2	3A	2	1	2
2A	2	1	2A	1	1	2
2A	2	1	2A	1	1	1
4	4A	3A	4	3	1	1
1B	2	0	1B	0	0	2
1A	1B	0	1A	0	0	1
3A	3	1	2B	1	2	2
1A	1A	0	1A	0	0	1
2A	3	0	2A	0	0	2
2B	2	1	2A	1	2	1
2B	3	2	3A	2	1	2
3A	3	3A	3B	3	2	2
4	4A	3B	4	3	2	2
3B	3	3A	3B	3	2	2
3B	4B	0	3B	0	0	2
3B	3	3A	3B	3	2	2
2B	3	2	3A	2	1	1
3C	4A	3A	3C	3	2	2
3B	3	3A	3B	3	2	1
3B	3	2	3A	2	2	2
2B	3	2	3A	2	1	2
3A	3	3A	3B	3	2	2
2A	2	1	2A	1	2	2
2A	3	0	2A	0	0	2
3A	3	3A	3B	3	2	2

3A	3	2	3A	2	1	1
1B	2	0	1B	0	0	2
1A	1B	0	1A	0	0	1
1A	1A	0	1A	0	0	1
3B	4A	1	3A	1	2	2
1A	1B	0	1A	0	0	2
2B	2	2	2B	2	1	2
4	3	3B	4	3	2	2
2B	3	1	2B	1	1	2
2A	3	0	2A	0	0	1
1A	1A	0	1A	0	0	1
2A	3	0	2A	0	0	2
3B	4A	1	3A	1	2	1
2B	2	1	2A	1	2	2
1B	1B	1	1B	1	1	2
3C	4B	3B	3C	3	2	2
1B	1B	1	1B	1	1	2
2B	2	3B	3A	3	2	2
2A	2	1	2A	1	1	2
2B	4A	0	2B	0	0	1
2A	3	0	2A	0	0	2
2B	3	1	2B	1	1	1
3C	4B	3B	3C	3	2	2
3C	4B	3B	3C	3	2	2
3B	3	3A	3B	3	2	2
2B	3	2	3A	2	1	2
4	4A	1	4	1	1	1
2A	2	1	2A	1	1	2
2B	3	1	2B	1	2	1
3C	4A	3B	3C	3	2	2
3A	3	2	3A	2	2	1
1A	1B	0	1A	0	0	1
3B	3	3B	3B	3	2	2
3C	4A	3B	3C	3	2	2
1A	1B	0	1A	0	0	1
2A	2	1	2A	1	1	2
2A	2	2	2B	2	1	2
3A	3	2	3A	2	2	2
4	4A	3B	4	3	2	2
4	4A	0	4	0	0	1
2A	2	1	2A	1	1	1
3B	4A	3A	3C	3	1	1
3C	4A	3A	3C	3	2	2
1B	2	0	1B	0	0	1
1A	1A	0	1A	0	0	2
3A	2	3B	3A	3	2	2
2B	3	1	2B	1	1	2
2A	2	1	2A	1	1	2
2B	3	2	3A	2	1	2
2A	3	0	2A	0	0	2
1A	1A	0	1A	0	0	2

3A	3	3A	3B	3	2	2
2B	3	2	3A	2	1	2
3C	4A	3A	3C	3	2	2
3B	3	3A	3B	3	2	2
2B	4A	0	2B	0	0	1
3A	3	3A	3B	3	1	2
1B	2	0	1B	0	0	2
3C	4B	3B	3C	3	1	2
4	4A	3B	4	3	2	2
2A	3	0	2A	0	0	2
4	4A	3B	4	3	2	1
1B	2	0	1B	0	0	1
3A	3	3A	3B	3	2	2
1A	1B	0	1A	0	0	2
1A	1B	0	1A	0	0	1
2A	2	1	2A	1	1	2
4	4B	3A	4	3	2	2
3B	3	3A	3B	3	2	1
3A	4A	1	3A	1	1	1
3C	4A	3B	3C	3	2	2
1B	1B	1	1B	1	2	1
4	3	3B	4	3	2	2
1B	2	0	1B	0	0	2
3A	3	2	3A	2	1	2
4	3	3B	4	3	2	2
3C	4A	3B	3C	3	2	2
4	4A	3B	4	3	2	2
3C	4A	3B	3C	3	2	2
3B	4A	2	3B	2	1	2
3C	4A	3B	3C	3	2	2
3A	4A	1	3A	1	1	2
3B	4A	1	3A	1	2	2
4	4A	3B	4	3	2	2
3A	4A	3A	3C	3	1	2
2B	3	2	3A	2	1	1
3A	3	3A	3B	3	1	2
4	4A	3B	4	3	2	2
3C	4A	3B	3C	3	2	2
3B	4A	3B	3C	3	2	2
2A	2	2	2B	2	1	2
2A	3	0	2A	0	0	2
2B	4A	0	2B	0	0	2
3C	4A	3A	3C	3	2	2
3A	3	2	3A	2	1	1
3C	4B	3B	3C	3	2	1
1A	1B	0	1A	0	0	1
3A	3	1	2B	1	2	1
2A	3	0	2A	0	0	2
3B	3	3B	3B	3	2	2
3C	4A	3A	3C	3	2	2
1A	1B	0	1A	0	0	1

1A	1B	0	1A	0	0	2
3A	3	2	3A	2	1	1
3C	4A	3A	3C	3	2	2
3B	4B	1	3B	1	2	1
3A	2	3A	3A	3	2	2
1A	1A	0	1A	0	0	2
2B	3	2	3A	2	2	2
1A	1B	0	1A	0	0	2
2A	3	0	2A	0	0	2
1A	1A	0	1A	0	0	2
4	4A	3B	4	3	2	2
3C	4A	2	3B	2	2	1
3A	3	2	3A	2	1	2
3A	4A	1	3A	1	2	1
3A	3	2	3A	2	1	2
4	4A	3A	4	3	1	2
1A	1A	0	1A	0	0	1
3B	3	2	3A	2	2	2
3B	3	3A	3B	3	2	2
1B	2	0	1B	0	0	2
4	3	3B	4	3	2	2
4	4A	3B	4	3	2	2
4	4B	3B	4	3	2	2
1A	1B	0	1A	0	0	2
1A	1B	0	1A	0	0	1
1A	1B	0	1A	0	0	2
2B	1B	2	2A	2	2	2
3C	4B	3A	3C	3	1	2
1B	2	0	1B	0	0	2
2B	4A	0	2B	0	0	2
2B	4A	0	2B	0	0	2
3A	3	2	3A	2	2	2
2A	3	0	2A	0	0	2
1B	2	0	1B	0	0	2
1A	1B	0	1A	0	0	2
2A	3	0	2A	0	0	2
2A	3	0	2A	0	0	2
3B	3	3B	3B	3	2	2
2A	3	0	2A	0	0	1
4	3	3A	4	3	2	1
3A	3	3A	3B	3	2	1
3C	4A	3A	3C	3	2	2
3C	4A	3B	3C	3	2	2
4	4A	3A	4	3	2	2
2B	3	1	2B	1	1	1
4	4B	3B	4	3	2	2
1A	1A	0	1A	0	0	1
1A	1B	0	1A	0	0	1
3A	3	2	3A	2	2	2
4	4A	0	4	0	0	1
2B	1B	3A	2B	3	2	2

1A	1B	0	1A	0	0	1
2B	3	2	3A	2	1	1
3C	4A	3A	3C	3	2	2
2A	2	1	2A	1	1	2
3C	4A	3A	3C	3	2	1
4	3	3B	4	3	2	2
4	3	3B	4	3	2	2
3B	3	3B	3B	3	2	2
1A	1B	0	1A	0	0	2
2B	4A	0	2B	0	0	2
3C	4A	3A	3C	3	2	2
3C	4A	3B	3C	3	2	2
2A	3	0	2A	0	0	2
1A	1B	0	1A	0	0	1
2B	2	2	2B	2	1	2
2B	4A	0	2B	0	0	1
3C	4A	3B	3C	3	2	2
3C	4A	3B	3C	3	2	1
2A	2	1	2A	1	1	1
1A	1A	0	1A	0	0	2
4	4A	3B	4	3	2	1
3A	3	2	3A	2	2	1
2A	2	1	2A	1	1	1
3A	4A	1	3A	1	1	1
1A	1A	0	1A	0	0	1
3B	4A	3A	3C	3	2	2
1A	1A	0	1A	0	0	2
2A	3	0	2A	0	0	1
1B	2	0	1B	0	0	1
2B	3	2	3A	2	1	2
1A	1B	0	1A	0	0	2
3C	4B	3A	3C	3	2	2
3A	3	3B	3B	3	2	1
4	4A	3B	4	3	2	2
4	4B	3B	4	3	2	2
2A	3	0	2A	0	0	2
1A	1B	0	1A	0	0	1
1A	1A	0	1A	0	0	2
3B	4A	2	3B	2	1	2
2B	3	2	3A	2	1	2
4	4B	3B	4	3	2	1
3B	4A	3A	3C	3	1	2
4	4A	3B	4	3	2	2
2B	2	2	2B	2	2	1
3C	4B	2	3C	2	2	2
3C	4A	3A	3C	3	2	2
3C	4A	3B	3C	3	2	1
3B	3	2	3A	2	2	1
3B	4A	3A	3C	3	1	2
1B	1B	1	1B	1	1	2
3C	4A	3B	3C	3	2	2

2B	3	1	2B	1	1	1
1B	2	0	1B	0	0	2
1B	2	0	1B	0	0	2
1A	1B	0	1A	0	0	2
2B	3	1	2B	1	1	2
4	3	0	4	0	0	1
2A	1B	2	2A	2	1	2
3B	4A	3B	3C	3	1	1
2B	2	2	2B	2	2	2
3A	4A	1	3A	1	2	2
2B	4A	0	2B	0	0	1
1A	1A	0	1A	0	0	2
1A	1A	0	1A	0	0	1
3C	4A	3B	3C	3	2	2
3B	3	3B	3B	3	2	2
2A	3	0	2A	0	0	2
2A	3	0	2A	0	0	2
2A	2	1	2A	1	1	1
3A	3	2	3A	2	1	2
2B	3	1	2B	1	2	2
1A	1B	0	1A	0	0	2
4	3	3A	4	3	2	1
3C	4A	3A	3C	3	2	2
2A	3	0	2A	0	0	2
3B	3	3A	3B	3	2	2
3C	4A	3A	3C	3	2	2
2B	3	1	2B	1	1	1
3B	4A	2	3B	2	1	1
1A	1B	0	1A	0	0	1
4	4A	3A	4	3	1	1
4	3	1	4	1	2	2
1A	1A	0	1A	0	0	1
3C	4A	3A	3C	3	2	1
3C	4A	3B	3C	3	2	2
4	4A	3B	4	3	2	2
3A	3	2	3A	2	2	2
3A	3	1	2B	1	2	2
2A	1B	2	2A	2	1	2
2B	3	2	3A	2	1	2
1B	2	0	1B	0	0	1
4	4A	3B	4	3	2	1
1B	1B	1	1B	1	1	2
2B	3	1	2B	1	1	1
3A	4A	3A	3C	3	1	2
